# Video summarization based on multi-scale feature fusion

**DOI:** 10.1371/journal.pone.0353773

**Published:** 2026-07-23

**Authors:** Jing Bao, Shipeng Xu, Jing Zhang

**Affiliations:** School of Cyberspace Security, Gansu University of Political Science and Law, Lanzhou, Gansu, China; Shanghai Jiao Tong University, CHINA

## Abstract

Video summarization aims to identify the important segments of a video and form a concise representation, enabling users to quickly grasp the core information. Existing graph-based video summarization methods suffer from insufficient modeling of multi-scale feature interactions and difficulties in balancing local and global features. To address this issue, this paper proposes a multi-scale feature fusion video summarization model based on the Message Passing Neural Network (MPNN) framework. First, the model extracts features of representative frames from video shots, and constructs a graph structure where shot features serve as nodes and inter-shot semantic similarity as edges. Second, it decouples the multi-scale features in the graph via the MPNN, and utilizes the Graph Attention Network (GAT) and Graph Neural Network (GNN) to extract local correlation features and global features, respectively. Finally, it fuses the original shot features, local features and global features as the final features to calculate shot importance and generate the video summary. On SumMe (F1 = 50.0) and TVSum (F1 = 61.8) datasets, MSF-MPNN achieves competitive performance against mainstream RNN/GNN-based methods. MSF-MPNN provides a practical solution for efficient video content extraction, with potential in video surveillance and short-video platforms.

## Introduction

The convergence of digital imaging technology and the internet has spawned massive volumes of video data, making efficient storage, intelligent browsing, and accurate retrieval core challenges in the field of computer vision. Video summarization technology provides an effective solution to this problem by extracting key information to generate concise representations, enabling users to quickly grasp the essence of videos [1–3]. Existing methods are roughly classified into two categories: static (keyframe-based) and dynamic (shot-based) summarization. Among them, dynamic summarization has garnered more attention due to its ability to integrate multimodal information (e.g., visual, audio, and textual information) [4–7].

Deep learning has driven the rapid development of the video summarization field. In particular, models such as Recurrent Neural Networks (RNNs) and Long Short-Term Memory (LSTM) networks have achieved notable progress in temporal modeling [8–11], but they still exhibit limitations in handling long-sequence dependencies and non-Euclidean data. The unique advantage of Graph Neural Networks (GNNs) in modeling node correlations and structural features has brought a new research perspective to the innovation of video summarization technology [10,12–15]. Although GNN-based methods have made some progress, they mostly focus on single-scale features and ignore multi-scale interactions as well as long-short temporal correlations, which limits these models’ ability to capture video semantics and leads to poor summary quality. Message Passing Neural Networks (MPNNs) integrate various existing graph neural network models (e.g., GNNs, GATs) into a general framework, update node embeddings through neighborhood aggregation, and thereby enable information propagation over graph structures [16,17]. They can enhance long-range information transmission and computational efficiency through various optimization strategies (e.g., introducing virtual edges, master nodes), realize feature complementarity and enhancement, and thus have unique advantages in multi-scale fusion tasks [18].

To address these issues, this paper proposes a multi-scale feature fusion video summarization model based on the MPNN framework. By combining semantic features of different scales in videos, such as shots, video clips, and complete videos, the model can more accurately capture key information and global structures in videos, thereby improving the evaluation accuracy of shot importance.

The main contributions of this paper can be summarized as follows:

1. It proposes a multi-scale graph construction and feature disentanglement strategy based on the MPNN framework to capture multi-scale features within shots, between shots, and at the video level;
2. It designs a cross-scale feature fusion module to dynamically aggregate multi-level semantic information;
3. It validates the accuracy and robustness of the proposed method on benchmark datasets.

## Related work

This section reviews research advances in video summarization and related work on Message Passing Neural Networks (MPNNs) and Graph Attention Networks (GATs).

### Video summarization

Deep learning-based video summarization techniques have achieved remarkable progress in modeling temporal and structural dependencies. Early methods mainly adopted Recurrent Neural Networks (RNNs) and Long Short-Term Memory (LSTM) networks to capture inter-frame sequential relationships. For instance, Zhang et al. [19] proposed an LSTM-based video summarization model, which utilized a temporal propagation mechanism to quantify frame importance and incorporated a diversity enhancement strategy to optimize keyframe selection. However, such methods have limitations in handling long-range dependencies in long videos and non-Euclidean data structures.

The emergence of Graph Neural Networks (GNNs) has gradually shifted video summarization techniques towards graph-structured modeling. Graph Convolutional Networks (GCNs) capture global structural dependencies through local spectral approximation [20]. Graph Attention Networks (GATs) assign adaptive attention weights to adjacent nodes, enhancing the model’s ability to focus on keyframes [21]. Zhong et al. [10] combined bidirectional LSTMs with Graph Attention Networks (GATs) to fuse semantic and visual features to reduce keyframe redundancy. Variational Graph Autoencoders (VGAEs) address this issue from the perspective of structural optimization by reconstructing adjacency matrices through learning the latent distribution of nodes, thereby reducing redundant connections [22]. Park et al. [23] proposed a recursive graph optimization strategy (SumGraph), which strengthened semantic correlations between nodes by iteratively optimizing the graph structure.

Feature fusion has emerged as an important direction for improving summary quality. For instance, Zhang et al. [24] fused original shot features with GAT-optimized structural features to improve the accuracy of shot importance evaluation; Zhang et al. [25] designed a dynamic fusion mechanism that combines shallow temporal features extracted by LSTM with deep structural features obtained through graph contrastive learning, effectively enhancing the semantic representation capability of features. Despite these advancements, systematic frameworks for modeling cross-scale interactions are still relatively limited.

In recent years, multimodal video analysis combining audio and visual information has become a key research area. Existing work indicates that audio signals have strong complementary properties in video saliency prediction, emotion matching and quality assessment [26–29]. With the rapid progress of video pre-training techniques, large-scale video pre-trained models (e.g., VideoMAE [30], CLIP [31]) are widely used for fine-grained visual feature extraction. As a mainstream optimization direction for video summarization, adaptive graph learning gradually replaces handcrafted similarity metrics, dynamically optimizing inter-shot adjacency weights via model losses to capture complex non-linear semantic relationships [32]. These advances offer valuable technical insights for multimodal extension and feature learning optimization of video summarization models.

### Message passing neural networks (MPNNs)

The Message Passing Neural Networks (MPNNs) proposed by Gilmer et al. [16] model graph-structured data through an iterative “message passing-aggregation-update” mechanism. They achieve global information integration by capturing correlations among local neighborhoods, subgraphs, and global structural features via hierarchical message passing. During their evolution, message generation has evolved from relying solely on node features in early models [33] to introducing attention mechanisms for dynamically assigning neighbor weights [21], and integrating edge features with multi-scale node states to enhance the hierarchy of message representations [34]. Neighborhood aggregation has advanced from simple summation or mean operations [20] to LSTM-based temporal aggregation [35], Set2Set-based ordered aggregation [16], and multi-scale neighbor sampling strategies [34]. Node update employs gating mechanisms or residual connections [36] to ensure the effective transmission of deep features. In this paper, by constructing video frames into a graph structure, we leverage MPNNs to capture inter-frame dependencies and utilize their multi-scale feature extraction ability to analyze content at different granularities, thereby generating more concise and accurate video summaries.

The semantic correlations between video shots are not strictly chronological and form a non-Euclidean structure. Temporally distant shots with similar content (e.g., echoing scenes) have strong semantic connections, while adjacent shots with abrupt content changes (e.g., shot transitions) show weak correlations. Traditional sequential models (RNN/LSTM) rely on regular temporal neighborhood assumptions and fail to capture such irregular high-dimensional relationships. The MPNN transforms non-Euclidean video data into a graph message passing framework, with shots as nodes and semantic similarity as edges. Its iterative message passing-aggregation-update mechanism enables information to spread over the semantic graph rather than being restricted to temporal neighborhoods, naturally fitting the non-Euclidean characteristics of video. During each iteration, nodes aggregate information from semantically similar neighbors and fuse local patterns with the global graph structure. This lays the theoretical foundation for using GAT and MPNN to extract local and global features respectively.

### Graph attention networks (GAT)

The Graph Attention Network (GAT) proposed by Veličković et al. [21] introduces a masked self-attention mechanism into graph structures. It dynamically learns neighbor weights to focus on key nodes, which significantly enhances the feature learning ability for non-Euclidean data and has become an important milestone in the development of graph neural networks. In the field of video summarization, the integration of GAT with temporal models has emerged as an important research direction. Zhong et al. [10] combined GAT with Bi-LSTM, transforming visual features into high-level representations through a Contextual Feature Transformation (CFT) mechanism and designing a Spatial Attention Model (SAM) to extract frame-level features, effectively reducing keyframe redundancy. After reconstructing the graph structure using Variational Graph Autoencoders (VGAEs), Zhang et al. [24] leveraged GAT to aggregate neighborhood information and fused original features with structural features to improve evaluation accuracy. Collectively, these works have constructed a technical foundation for applying GAT in video summarization, ranging from basic modeling to complex scenario applications, and have provided valuable insights for multi-scale feature fusion.

## Method

**Fig 1:**
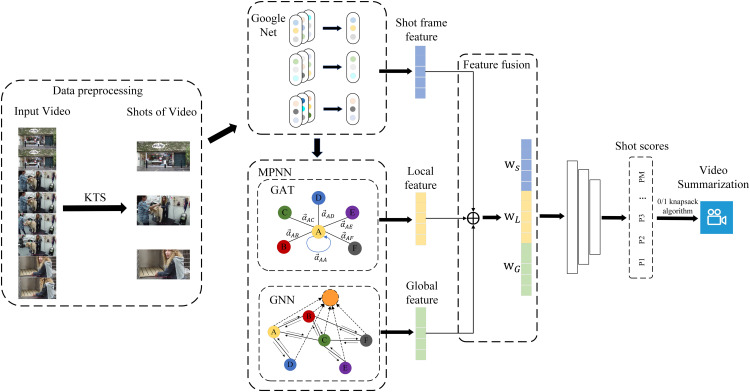
Network architecture for video summarization based on multi-Scale feature fusion.

As shown in Fig 1, this paper proposes a multi-scale feature fusion video summarization model based on the Message Passing Neural Network (MPNN) framework to extract and learn multi-level semantic features of videos. The model comprises four key components: feature extraction, feature learning, feature fusion, and shot selection. In the feature extraction component, the input video is first segmented into multiple shots, and representative frame features of each shot are extracted via the GoogLeNet architecture as shot-level representations. In the feature learning component, an undirected graph is constructed with shot features as nodes and inter-shot similarity as edges. The MPNN framework is then employed to decouple multi-scale features within the graph. Local features are extracted by aggregating neighborhood information of adjacent shot nodes via the Graph Attention Network (GAT), while global features which capture the temporal and spatial structure of the entire video, are extracted through global modeling and message passing via the Graph Neural Network (GNN). In the feature fusion component, multi-scale features are integrated to enhance the hierarchical representation ability of node features. In the shot selection component, importance scoring is estimated based on the fused shot node features, and the key shots are selected to generate the final video summary.

### Feature extraction

For the input raw video, it was first defined as a sequence of frames $F=\{f_{n}{\}}_{n=1}^{N}$, where $f_{n}$ denotes the n-th frame of the video with dimensions w×h×3 (*w* and *h* denote the width and height of the frame, while 3 denotes the number of RGB channels), and *N* denotes the total number of frames of the video. The KTS algorithm [37] was employed to segment the input video into shots, yielding a set of shots $S=\{s_{m}{\}}_{m=1}^{M}$, where $s_{m}$ denotes the m-th shot comprising $T_{m}$ frames and such that $N=\sum_{m=1}^{M}T_{m}$ (*M* denotes the total number of shots). For each shot, GoogLeNet [38] was utilized to extract frame-level deep features, forming a feature set $G=\{g_{m}{\}}_{m=1}^{M}$, where $g_{m}$ denotes the feature matrix of the m-th shot with dimensions $D\times T_{m}$ (*D* denotes the feature dimension).

### Feature learning

#### Graph construction.

To model the semantic correlations between video shots, a graph structure is constructed in which the representative frame features of each shot serve as nodes. The node set comprises representative frame features of each shot, denoted as $X=\{x_{1},x_{2},\ldots ,x_{M}\}$ (where $x_{i}$ represents the feature vector of the representative frame of the i-th shot, with dimension D×1, and M denotes the total number of shots). The edge weights are represented by an adjacency matrix $A\in {\mathbb{R}}^{M\times M}$, which reflects the semantic similarity between distinct shot features. The representative frame of each shot is selected as the frame with the smallest average Euclidean distance to all other frames within the same shot [25]. Its representativeness score is computed as follows:


$$\begin{array}{c} r_{m}=\exp\left(-\frac{1}{T_{m}}\sum_{t=1}^{T_{m}}\min_{t^{\prime }\in Y}\|x_{t}-x_{t^{\prime }}\|\right) \end{array}$$ (1)

where *Y* denotes the set of frames within shot *m*.

The min operation selects the frame within each shot that has the minimum average Euclidean distance to all other frames, namely the medoid of the shot. Compared with random selection and first-frame selection strategies, the medoid is insensitive to variations in frame distribution inside a shot. It can stably represent the overall visual content of the shot and further improve the quality of graph node features. The exponential function maps the original distance, whose range is [0,+∞), to the interval (0, 1]. A smaller distance corresponds to a score closer to 1, which means higher confidence for the representative frame. Owing to the exponential decay property, frames with large distances yield scores that rapidly approach zero. This effectively suppresses the interference caused by outlier frames and enables representative frames to concentrate on dense regions of shot features. Meanwhile, this function maintains the monotonicity of distance values, which facilitates cross-shot comparisons in subsequent steps.

When constructing the adjacency matrix, cosine similarity is employed to compute edge weights [39], which can more accurately reflect node similarity in comparison with the dot product method, the Gaussian method, and the concatenation method [40]. The edge weights are computed as follows:


$$\begin{array}{c} e_{ij}=\frac{{x_{i}}^{T}x_{j}}{\|x_{i}{\|}_{2}\cdot \|x_{j}{\|}_{2}} \end{array}$$ (2)

where $e_{ij}$ denotes the (*i*,*j*)-th element of the adjacency matrix, and $\|x_{i}{\|}_{2}$ denotes the L2 norm of $x_{i}$. The resulting graph structure is denoted as *G* = (*X*, *E*), where the edge set $E=\{e_{ij}|1\leq i,j\leq M\}$.

In this work, we employ GoogLeNet to extract shot features and construct a static adjacency matrix via fixed cosine similarity. This design is intended to control experimental variables, as standardized pipelines for feature extraction and graph construction rule out performance variations introduced by preprocessing, allowing us to precisely quantify the effectiveness of the proposed multi-scale fusion module. Since our core innovation focuses on the multi-scale fusion mechanism rather than feature extraction or graph design, we adopt the standard configurations widely used in the community to ensure fair comparison with existing baselines.

However, this manually defined paradigm has inherent drawbacks. All edge weights are frozen during model training and cannot be dynamically updated to adapt to diverse video semantics. For videos with complex scenes and broad semantic ranges, this approach struggles to capture latent shot correlations, making it less competitive than state-of-the-art adaptive graph learning methods. To mitigate this issue, we leverage the GAT attention mechanism to dynamically tune aggregation weights on the static graph, and the iterative message passing of MPNN allows information to spread across multi-hop neighbors, partially overcoming the constraints of predefined edges. Even so, fully adaptive graph learning remains for future exploration.

#### Local feature learning.

In video summarization, shot importance is determined by both its intrinsic content and semantically related neighboring shots. For instance, a sequence of similar consecutive shots often depicts a single key event. Due to the non-Euclidean nature of video data, shots with similar semantics are not always temporally adjacent. The Graph Attention Network (GAT) utilizes the attention mechanism to compute pairwise semantic similarities between shots and aggregate neighboring nodes with adaptive weights. It explicitly models local semantic correlations and enables the model to focus on semantically relevant shots, strengthening fine-grained feature representation. If local correlations are not modeled, the model fails to distinguish accidental adjacency from semantic relevance and tends to generate redundant summaries. Accordingly, we employ GAT to dynamically aggregate neighborhood information and assign differentiated weights to neighboring nodes, which effectively learns local node features and boosts feature discriminability and robustness.

To effectively capture local features of nodes in the graph, the Graph Attention Network (GAT) is employed to aggregate information from neighboring nodes through an attention mechanism. By assigning adaptive attention weights to neighboring nodes with varying importance, the discriminability and robustness of node features are further improved.

First, the attention scores between a target node and its neighboring nodes are computed. After mapping node features to a high-dimensional space using a linear transformation matrix $W\in {\mathbb{R}}^{D\times D^{\prime }}$, an attention vector α (with a dimension twice the size of the mapped features) is generated by a Multi-Layer Perceptron (MLP). The attention scores are computed using feature concatenation followed by a LeakyReLU activation, as shown in the formula:


$$\begin{array}{c} e_{ij\text{-GAT}}=\mathrm{LeakyReLU}\left(\alpha^{T}\left[Wx_{i}\|Wx_{j}\right]\right) \end{array}$$ (3)

where $\left[Wx_{i}\|Wx_{j}\right]$ denotes the concatenation of the mapped target node feature $Wx_{i}$ and the mapped neighboring node feature $Wx_{j}$, and $e_{ij\text{-GAT}}$ represents the importance of the *j*-th neighboring node to the *i*-th target node.

To highlight key neighborhood information, the softmax function is employed to normalize the attention scores, as follows:


$$\begin{array}{c} {\bar{e}}_{ij}=\mathrm{softmax}\left(e_{ij\text{-GAT}}\right)=\frac{\exp\left(e_{ij\text{-GAT}}\right)}{\sum_{k\in N_{i}}\exp\left(e_{ik\text{-GAT}}\right)} \end{array}$$ (4)

where $N_{i}$ denotes the neighborhood set of node *i*. Finally, the local feature of node *i* is obtained by performing a weighted summation of neighboring node features with the normalized attention scores ${\bar{e}}_{ij}$, forming a more discriminative local feature representation.

#### Global feature learning.

Local features capture semantic shot correlations, yet they fail to effectively characterize long-range spatio-temporal dependencies among segments separated by multiple shots but sharing similar semantics. With no direct edges between such shots, single-layer neighborhood aggregation cannot enable information exchange. Single-round message passing is limited to one-hop neighbor interaction. In contrast, MPNN leverages iterative message passing and aggregation. After multiple rounds of updates, each node continuously receives feature information from indirect neighbors and gradually fuses spatio-temporal features of distant shots. Without explicit global structure modeling, the model loses the overall narrative structure and produces fragmented summaries. To address this, we adopt MPNN’s message passing mechanism for global feature learning. The MPNN framework consists of three core components: message passing, feature update, and global aggregation. MPNN-derived global features complement GAT-based local features, endowing the model with both fine-grained semantic sensitivity and global structure perception.

In the message passing phase, for graph *G* = (*X*, *E*) (where *X* denotes the node feature matrix and *E* denotes the edge set), node *i* receives messages from its neighborhood $N_{i}$ (defined by non-zero elements in *E*). First, the features of node *i* and its neighboring node *j* are concatenated into $\left[x_{i},x_{j}\right]$, and a single message is generated via a message network (a linear layer). Then all neighborhood messages are summed up to derive the total message for node *i*. The formula is as follows:


$$\begin{array}{c} m_{i}=\sum_{j\in N_{i}}\mathrm{Linear}\left([x_{i},x_{j}]\right) \end{array}$$ (5)

If a node has no neighbors ($N_{i}=\emptyset$), the message $m_{i}$ is set to a zero vector 0.

In the feature update phase, a Gated Recurrent Unit (GRU) is employed to integrate the original feature of node *i* with the received message for feature update:


$$\begin{array}{c} x_{i}^{\prime }=\mathrm{GRU}\left(m_{i},x_{i}\right) \end{array}$$ (6)

The GRU dynamically balances the contributions of the original node feature and the received message through a gating mechanism, enhancing the temporal consistency of the node feature.

In the global aggregation phase, all updated node features $\left\{x_{i}^{\prime }\right\}$ are aggregated to generate graph-level global features, with support for various aggregation strategies: summation, averaging, maximum pooling, attention mechanism, or LSTM. Since LSTM excels at modeling temporal information, the resulting global features better align with the temporal evolution of video content. Thus LSTM is selected as the global aggregation strategy. The updated node features are fed into the LSTM in the order of the original video shots, and the aggregated features are mapped to the target dimension via a linear layer to generate global feature *g* that can capture both the spatial correlations and temporal dependencies of video shots:


$$\begin{array}{c} g=\mathrm{Linear}\left(\mathrm{LSTM}\left(x_{1}^{\prime },x_{2}^{\prime },\ldots ,x_{M}^{\prime }\right)\right) \end{array}$$ (7)

where $g\in {\mathbb{R}}^{d}$ and *d* denotes the output dimension. This global feature integrates the correlation information of all nodes and captures the overall structural characteristics of the video.

### Feature fusion

To fully preserve the multi-dimensional information of video shots, this paper fuses multi-scale features to construct comprehensive features with both the capability of detailed representation and global correlation. Fusing feature information from distinct network levels not only preserves the intrinsic semantic integrity of video shots but also captures richer local and even global contextual dependencies. This is critical for enhancing the discriminative power of shot features and thus improving the accuracy of shot importance evaluation [41]. Three types of features are involved in the fusion process: the first type corresponds to the original deep features *G* of shots, comprising representative frame features extracted via the GoogLeNet architecture, which directly capture the visual semantic information of shots; the second type refers to the graph-based local features *L*, acquired through dynamically weighted aggregation of neighboring node features via the GAT, which describe the local dependency relationships between a shot and its surrounding shots; the third type is the graph-based global feature vector *g*, generated via MPNN message passing and global aggregation mechanisms, which captures the overall structural pattern of the video shot graph network.

Feature fusion employs a combination of concatenation and the gating mechanisms. First, the three types of features are concatenated dimension-wise into a high-dimensional feature vector $F_{\text{cat}}=[G\|L\|g]$ to fully preserve the original information of each feature. Subsequently, a gating unit is introduced to perform adaptive filtering on the concatenated features, and gating coefficients are learned by optimizing the parameter vector $W_{g}$ and bias $b_{g}$:


$$\begin{array}{c} \gamma =\sigma \left(W_{g}\cdot F_{\text{cat}}+b_{g}\right) \end{array}$$ (8)

where σ(·) denotes the sigmoid activation function, and γ serves to dynamically adjust the contribution weights of each feature component. The final fused feature is obtained as follows:


$$\begin{array}{c} F=\gamma ⊙F_{\text{cat}} \end{array}$$ (9)

The fused feature *F* is fed into a Multi-Layer Perceptron (MLP), which outputs the predicted shot importance scores *S*_pred_ via non-linear transformation:


$$\begin{array}{c} S_{\text{pred}}=\mathrm{MLP}\left(F\right) \end{array}$$ (10)

### Loss function

The model employs the Mean Squared Error (MSE) loss function to measure the discrepancy between predicted values and ground truth values. As a commonly used loss function for regression tasks, MSE improves the prediction accuracy by minimizing the squared differences between the predicted and ground truth values. The formula of the loss function is as follows:


$$\begin{array}{c} {ℒ}_{\text{MSE}}=\frac{1}{M}\sum_{i=1}^{M}{\left(S_{\text{real}}^{\left(i\right)}-S_{\text{pred}}^{\left(i\right)}\right)}^{2} \end{array}$$ (11)

where *M* denotes the total number of shots, $S_{\text{real}}^{\left(i\right)}$ denotes the ground-truth score of the *i*-th shot, and $S_{\text{pred}}^{\left(i\right)}$ denotes the predicted score of the *i*-th shot.

## Experiments

This section presents the benchmark datasets, evaluation metrics, and experimental results of the proposed model for video summarization.

### Datasets

This study employs two publicly available benchmark datasets widely used for video summarization tasks, namely SumMe [42] and TVSum [43], to evaluate the performance of the proposed model.

SumMe contains 25 user-generated videos covering various scenarios, including sports activities, daily events, and documentaries. The videos have an average duration of around 146 seconds. Each video is annotated with frame-level importance labels by 15–18 annotators, distinguishing key frames (labeled 1) from non-key frames (labeled 0) in a binary manner.

TVSum consists of 50 videos from the YouTube platform, including news reports, sports events, and teaching tutorials, with an average duration of around 235 seconds. A 1–5 point rating scale is adopted for this dataset, where 20 users independently rate the importance of each frame, and the final average score serves as the quantitative measure of frame-level importance.

Since the above-mentioned datasets are annotated at the frame level, whereas this study adopts shots as the processing unit, the KTS algorithm was employed for video shot segmentation. The importance score of each shot is obtained by aggregating the annotated frame-level scores within the shot. The 0–1 knapsack algorithm was then applied to generate the final video summaries. In the experiments, each dataset was randomly split into an 80% training set and a 20% test set. K-fold cross-validation was adopted to ensure the robustness of the experimental results and reduce the impact of data split bias on model evaluation.

### Evaluation metrics

This study employs the F1-score, which is widely used in video summarization tasks, as the core evaluation metric. The F1-score is calculated as the harmonic mean of Precision and Recall, providing a comprehensive measure of the model’s performance in key content extraction. Precision measures the accuracy of the generated summary and is defined as the ratio of matching frames to the total number of frames selected by the model. Recall measures the ability to cover important content and is defined as the ratio of matching frames to the total number of manually annotated key frames. The specific calculation formulas are as follows:


$$\begin{array}{c} \text{Precision}=\frac{N_{\text{match}}}{N_{\text{extract}}} \end{array}$$ (12)

$$\begin{array}{c} \text{Recall}=\frac{N_{\text{match}}}{N_{\text{US}}} \end{array}$$ (13)

$$\begin{array}{c} \text{F1}=\frac{2\times \text{Precision}\times \text{Recall}}{\text{Precision}+\text{Recall}} \end{array}$$ (14)

where *N*_match_ denotes the number of frames in the generated summary that match manual annotations, *N*_extract_ denotes the total number of frames extracted by the model, and *N*_US_ denotes the total number of manually annotated key frames.

In addition to the F1-score, we adopt Kendall’s τ and Spearman’s ρ to evaluate the ranking consistency between the predicted shot importance scores and the ground truth. These rank correlation coefficients measure the monotonic relationship between two rankings, with higher values indicating better agreement.

### Results

This section first compares the proposed model with RNN-based, GNN-based, and other state-of-the-art video summarization approaches, then evaluates the effectiveness of each module through ablation studies, and finally presents visualizations of the experimental results.

### Comparative experiments

Table 1 presents the comparison results between the proposed method and other state-of-the-art methods on the two benchmark datasets. Specifically, vsLSTM employs a bidirectional LSTM to capture inter-frame long-range temporal dependencies and outputs frame-level importance scores via an MLP. dppLSTM further incorporates a Determinant Point Process (DPP) to improve the diversity of key frames. SUM-GAN is an unsupervised adversarial LSTM-based framework that optimizes summary quality through adversarial training. CSNetsup addresses the flat distribution of output scores by designing a variance loss, constructs a Chunk and Stride Network (CSNet) to fuse local and global temporal views, and introduces a differential attention mechanism to capture dynamic scene transitions. HSA-RNN employs two layers of bidirectional LSTMs to mitigate the gradient vanishing problem induced by long sequences and capture inter-shot temporal dependencies. FCSN replaces recurrent models with a fully convolutional network and expands the receptive field by stacking convolutional layers to capture long-range temporal dependencies. RSGN employs an LSTM to encode frame-level temporal dependencies, a Graph Convolutional Network (GCN) to capture shot-level global dependencies, and incorporates a reconstructor to assess the summary’s ability to preserve original video content and inter-shot dependencies. GAT adjusted Bi-LSTMunsup extracts frame-level visual features via the Spatial Attention Model (SAM) and employs GAT to optimize the selection probabilities of key frames. GCANsup comprises two components: embedding learning and context fusion, which fuses the output information flows of the temporal and graph branches to generate contextual representations of video frames for computing their importance scores. VOGNet employs a Variational Graph Autoencoder (VGAE) to reconstruct the graph structure for redundant information removal, and subsequently optimizes the graph structure via GAT. DGC-FNet extracts video temporal and structural features via stacked L-G Blocks, and optimizes the graph structure through graph contrastive learning to mitigate redundancy. TAMGCN adopts an adaptive graph convolutional network with dynamic adjacency matrices, leveraging the attention mechanism to capture dynamic dependencies between shots. VideoSAGE formulates video summarization as a graph-based binary node classification task and directly optimizes for Kendall’s τ. LGRLN utilizes language-guided graph representation learning by building forward, backward and undirected graphs, and integrates dual-threshold graph convolution with cross-modal language embedding to generate accurate summaries.

**Table 1:** Performance comparison with existing advanced methods.

Model	SumMe			TVSum		
	F1	τ	ρ	F1	τ	ρ
vsLSTM [19]	37.6	0.04	0.06	54.2	0.04	0.05
DPP-LSTM [19]	38.6	−0.03	−0.03	54.7	0.03	0.04
SUM-GAN [11]	41.7	−0.01	−0.01	56.3	−0.05	−0.07
CSNetsup [44]	48.6	–	–	58.5	–	–
HSA-RNN [8]	44.1	0.06	0.07	59.8	0.08	0.09
FCSN [45]	48.8	–	–	58.4	–	–
RSGN [13]	45.0	0.08	0.09	60.1	0.08	0.09
GAT adjusted Bi-LSTMunsup [10]	51.5	–	–	59.1	–	–
GCANsup [46]	53.0	–	–	60.7	–	–
VOGNet [24]	49.8	–	–	60.8	–	–
DGC-FNet [25]	51.5	–	–	60.3	–	–
TAMGCN [32]	53.2	–	–	60.8	–	–
VideoSAGE [47]	46.0	0.12	0.16	58.2	0.30	0.42
LGRLN [48]	54.7	0.14	0.19	58.3	0.30	0.43
**MSF-MPNN (ours)**	**50.0**	**0.08**	**0.10**	**61.8**	**0.27**	**0.37**

For baseline methods, Kendall’s τ and Spearman’s ρ values are taken from the respective original papers or public literature; “-” indicates that the original paper did not report the value.

The experimental results (Table 1) indicate that the proposed multi-scale feature fusion method (MSF-MPNN) achieves competitive performance on both benchmark datasets (SumMe and TVSum). Specifically, MSF-MPNN yields notable improvements over traditional RNN-based methods. Compared to vsLSTM and DPP-LSTM, MSF-MPNN improves the F1-score by 12.4% and 11.4% on SumMe, and by 7.6% and 7.1% on TVSum, respectively. These improvements can be attributed to the capability of the multi-scale graph structure to model long-range dependencies and capture non-Euclidean structural relationships. Compared with SUM-GAN, the unsupervised generative framework based on adversarial training, MSF-MPNN improves the F1-score by 8.3% (SumMe) and 5.5% (TVSum), demonstrating the superiority of supervised multi-scale feature fusion in key information extraction.

Compared with mainstream GNN-based methods, the performance gains of MSF-MPNN are more targeted. On the SumMe dataset, while its F1-score is slightly lower than those of GCANsup, GAT-adjusted Bi-LSTMunsup, and DGC-FNet, it is significantly higher than that of RSGN (which solely relies on GCN to capture global dependencies) and VOGNet (which only reconstructs the graph structure via VGAE). This finding indicates that the fusion strategy that dynamically balances local and global features is more effective than strategies that rely on single-scale graph feature representation. On the TVSum dataset, MSF-MPNN achieves the highest F1-score among all comparative methods, confirming the effectiveness of the multi-scale feature disentanglement and dynamic weight adjustment mechanism.

Further comparison with recent graph neural network-based methods shows that MSF-MPNN achieves competitive performance. Against TAMGCN, MSF-MPNN improves the F1-score by 1.0% on TVSum and is 3.2% lower on SumMe. Against VideoSAGE, MSF-MPNN outperforms it by 4.0% and 3.6% on the two datasets, respectively. When compared with LGRLN, MSF-MPNN surpasses it by 3.5% on TVSum while trailing by 4.7% on SumMe.

From the perspective of ranking quality, MSF-MPNN also performs robustly. On the TVSum dataset, the Kendall’s τ (0.27) and Spearman’s ρ (0.37) of MSF-MPNN are significantly higher than those of vsLSTM (τ=0.04, ρ=0.05), DPP-LSTM (τ=0.03, ρ=0.04) and RSGN (τ=0.08, ρ=0.09). Meanwhile, the differences between MSF-MPNN and VideoSAGE (τ=0.30, ρ=0.42) as well as LGRLN (τ=0.30, ρ=0.43) are also within an acceptable range.

The core contributor to the performance gains is the in-depth modeling of dynamic interactions among multi-scale features. The fine-grained graph representation learned through GAT captures local semantic correlations between shots, effectively mitigating the limitation of RNN-based or GCN-based models in insufficiently weighting neighborhood information. The coarse-grained graph representation constructed via MPNN models video-level global dependencies, compensating for the inability of local features to capture long-range temporal structures. The dynamic weight adjustment mechanism adaptively balances the contributions of features at different scales, thereby improving both the accuracy and coverage of key content extraction.

### Ablation experiments

To validate the effectiveness of each core component of the proposed method, we designed six ablation variants and evaluated their performance on the SumMe and TVSum datasets. All variants adopted an identical feature extraction pipeline and training protocol to ensure experimental fairness. The six ablation variants are denoted as Raw, GAT, MPNN, Raw + GAT, Raw+MPNN, and the full model MSF-MPNN. Specifically, Raw comprises only a Multi-Layer Perceptron (MLP) and directly uses the raw shot features as input for importance scoring; GAT uses only local features extracted by the Graph Attention Network; MPNN uses only global features extracted by the Message Passing Neural Network; Raw + GAT and Raw+MPNN adopt concatenated features combining raw shot features with local or global features, respectively; the full model MSF-MPNN fuses raw features, GAT-based local features, and MPNN-based global features through a gated mechanism.

The experimental results are shown in Table 2. Among all single-feature variants, the Raw variant outperforms the variants using only GAT or MPNN on both datasets. This indicates that the pre-trained GoogLeNet features possess strong semantic representation capability, while standalone graph structure learning tends to cause performance degradation due to over-smoothing. For dual-feature fusion variants, the performance of Raw + GAT and Raw+MPNN is roughly comparable to that of the Raw model (SumMe: 45.0% and 45.5%, TVSum: 60.9% and 60.6%, respectively). This demonstrates that simply introducing local or global features cannot bring effective performance gains, and feature redundancy may instead limit the representation ability of the model. Specifically, the performance of Raw + GAT is slightly lower than the Raw baseline. When modeling merely with local neighborhood information, redundant similar edges in the graph lead to scattered attention and increased feature noise. Even with the introduction of raw shot features, the absence of global semantic constraints fails to effectively suppress the scattered attention caused by invalid neighbors. In contrast, the complete model MSF-MPNN further incorporates MPNN global features on the basis of Raw + GAT. It effectively suppresses the negative impacts of redundant edges by virtue of global temporal constraints, and finally achieves the optimal performance (SumMe: 50.0%, TVSum: 61.8%), with improvements of 4.7 and 0.7 percentage points over the Raw model, respectively. Further analysis confirms the complementary nature of the three feature types, and only their fusion can fully exploit the advantages of different scales.

**Table 2:** F1-score results of ablation experiments on the SumMe and TVSum datasets.

Model	Features			SumMe	TVSum
	RAW	Local	Global		
Raw	✓			45.3	61.1
GAT		✓		41.3	56.8
MPNN			✓	40.6	56.7
Raw + GAT	✓	✓		45.0	60.9
Raw + MPNN	✓		✓	45.5	60.6
MSF-MPNN (Full)	✓	✓	✓	50.0	61.8

**Fig 2:**
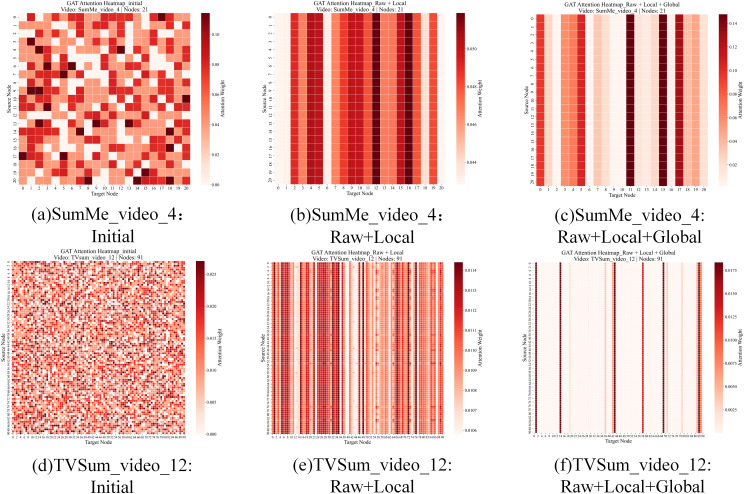
Attention heatmap comparison between Raw + GAT and the full MSF-MPNN model on representative videos from SumMe and TVSum.

Fig 2 presents ablation attention heatmaps of two representative videos from the SumMe and TVSum datasets. (a) and (d) illustrate the attention weight distribution at the model initialization stage for SumMe_video_4 and TVSum_video_12, respectively. At this stage, the attention parameters have not been trained, and the weights form an irregular distribution generated by random initialization, serving only as a baseline reference. (b) and (e) show the attention weight distribution of the corresponding videos under the Raw + GAT model. Since this model only adopts raw features and local GAT features and lacks global semantic constraints, it is disturbed by redundant similar edges in the graph. Consequently, the attention weights are scattered, and numerous non-key shots are assigned high weights, leading to obvious attention divergence. (c) and (f) display the attention results of the complete MSF-MPNN model. After introducing MPNN global features for multi-scale fusion, the model benefits from global video content constraints. Its attention is clearly focused on core key shots, and the attention weights corresponding to redundant neighborhoods are greatly suppressed. The visualized patterns are consistent across both datasets, intuitively verifying that global feature constraints are the key factor for reversing the negative impacts of redundant edges and achieving performance improvement.

Combined with the heatmap visualization results, the above experimental metrics jointly verify the effectiveness of the proposed multi-scale feature fusion scheme. The GAT module leverages adaptive weighting to explore local semantic correlations between shots and enhance fine-grained representation. The MPNN module models global long-range temporal dependencies via iterative message passing and provides global semantic constraints. After the adaptive gating fusion of raw visual features, local GAT features and global MPNN features, the model can effectively alleviate attention divergence and feature degradation caused by redundant neighborhoods, which are common drawbacks of models relying merely on local features. This realizes the complementation of multi-scale information and ultimately improves the accuracy of shot importance scoring as well as the overall quality of video summarization.

### Visualization results

**Fig 3:**
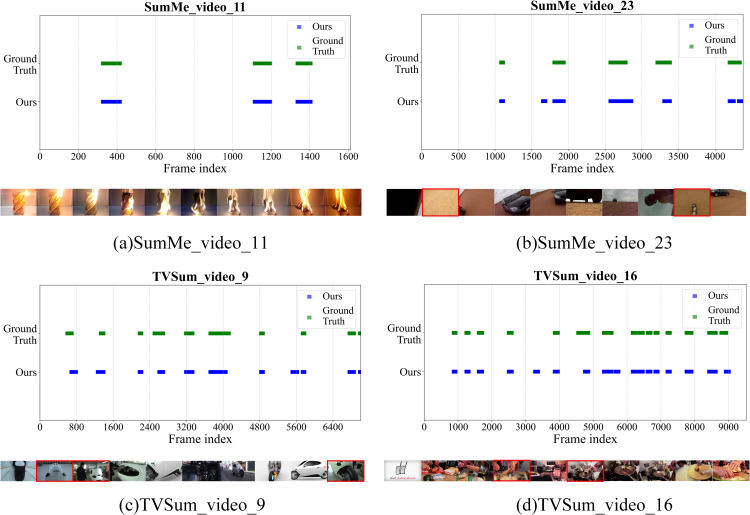
Visualization comparison between the model-generated summaries and the ground truth.

To visually illustrate the performance of the proposed multi-scale feature fusion method, we visualize the summaries generated by the model and analyze representative video samples from the SumMe and TVSum datasets. Fig 3 presents the comparative results between the model-generated summaries and the manual annotations using Video 11 and Video 23 from SumMe, and Video 9 and Video 16 from TVSum. The key frames generated by MSF-MPNN exhibit high consistency with the manual annotations, confirming the model’s ability to accurately capture the core content of videos. Specifically, the ground truth labels in the SumMe dataset are provided in a binary format that distinguishes key frames from non-key frames, resulting in a relatively sparse distribution. In contrast, the TVSum dataset adopts a 1–5 rating scale, providing more fine-grained annotations and yielding a denser label distribution. The visualization results further show that the summaries generated by MSF-MPNN are closely aligned with the ground truth labels, and the model can accurately identify representative frames across various scenarios. This validates the model’s adaptability to different label densities and scenario types.

## Discussion and conclusion

To address the insufficient modeling of multi-scale feature interactions and the difficulty in balancing local and global features in existing graph-based video summarization methods, this paper proposes a multi-scale feature fusion model for video summarization based on the Message Passing Neural Network (MPNN) framework. The model first segments the video into shots and extracts representative frame features. It then constructs a graph in which shot features serve as nodes and inter-shot similarities define the edges. It then decouples and learns both local correlation features and global structural features through the MPNN framework. Finally, the original shot features, local features, and global features are fused to compute shot importance scores and generate the final summaries. Experimental results on the benchmark SumMe and TVSum datasets demonstrate that the proposed model achieves competitive F1-scores compared to mainstream baseline methods, effectively enhancing the accuracy of shot importance assessment. Future research will focus on the following four optimization directions. First, we will replace GoogLeNet with fine-grained pre-trained feature extraction networks, e.g., large-scale video pre-trained models such as VideoMAE and CLIP. We will also adopt adaptive graph learning strategies to iteratively and dynamically optimize the adjacency matrix according to model training losses, overcoming the limitations of fixed cosine similarity and further improving the capability to mine key video content in complex scenarios. Second, we will integrate multimodal information including vision, audio, and text into the graph network framework. Third, we will explore privacy-preserving mechanisms for video summarization. Finally, we will extend our model to augmented and transfer settings to systematically evaluate its cross-domain generalization capability.
